# Simultaneous mapping of temporally-resolved blood flow velocity and oxygenation in femoral artery and vein during reactive hyperemia

**DOI:** 10.1186/1532-429X-13-66

**Published:** 2011-10-28

**Authors:** Michael C Langham, Felix W Wehrli

**Affiliations:** 1 Laboratory for Structural NMR Imaging, Department of Radiology, University of Pennsylvania Medical Center, Philadelphia, PA, USA

## Abstract

**Background:**

Post-occlusive hyperemia is often used as a paradigm to evaluate vascular reactivity, for example by measuring post-ischemic flow-mediated dilation, arterial blood flow or temporally resolved venous blood oxygenation (HbO_2_). Here we demonstrate the feasibility of a simultaneous measurement of blood flow and HbO_2 _in the femoral circulation as part of a single procedure.

**Methods:**

A multi-echo GRE pulse sequence was designed and implemented to collect velocity-encoded projections in addition to full-image echoes for field mapping as a means to quantify intravascular magnetic susceptibility. The method's feasibility was evaluated at 3T in a small pilot study involving two groups of healthy subjects (mean ages 26 ± 1.6 and 59 ± 7.3 years, N = 7 and 5, respectively) in terms of six parameters characterizing the time-course of reactive hyperemia and their sensitivity to differentiate age effects. The reproducibility was assessed on two of the seven young healthy subjects with three repeated measurements.

**Results:**

The physiological parameters agree with those obtained with current methods that quantify either velocity or HbO_2 _alone. Of the six measures of vascular reactivity, one from each group was significantly different in the two subject groups (p < 0.05) even though the study was not powered to detect differences. The mean coefficient of variation (CV) from two subjects undergoing repeat scans were approximately 8% for the oximetric and the arterial velocimetric parameters in the femoral vein and artery, respectively, considerably below intersubject CVs (20 and 35%, for the young and older subject groups, respectively).

**Conclusion:**

The proposed method is able quantify multiple parameters that may lead to more detailed assessment of peripheral vascular reactivity in a single cuff paradigm rather than in separate procedures as required previously, thereby improving measurement efficiency and patient comfort.

## Background

In the United States, cardiovascular diseases (CVD) account for more deaths than cancers, chronic obstructive pulmonary diseases and accidents combined [1]. The burden of CVD in the future is expected to grow due to the aging population, prevalence of obesity [2,3] and unhealthy lifestyle choices that include smoking [4], lack of physical activity [5] and poor nutrition [6,7]; all factors predisposing subjects to develop systemic atherosclerosis that can remain silent for decades. The earliest stage of pathogenesis of atherosclerosis, the most common cause of CVD, is endothelial dysfunction (EDF), which can manifest as impaired reactivity in the peripheral vascular bed (e.g. brachial or femoral arteries) due to the suppression of nitric oxide (NO) [8] synthesis. In normal vascular function greater demand for blood flow in response to a physiological challenge is accommodated initially via reduction of microvascular resistance. Subsequently, increase in shear stress to the vessel wall promotes NO release resulting in vasodilation (referred to as flow-mediated dilation (FMD)) of the conduit artery [9]. Thus, micro- and macrovascular function can be assessed during reactive hyperemia.

Among non-invasive imaging modalities used to quantify reactive hyperemia in peripheral arteries are near-infrared spectroscopy (NIRS) [10-13], single photon emission computed tomography [14], cardiovascular magnetic resonance (CMR) [15-18], and ultrasound [19]. NIRS and ultrasound (US) are most commonly used due to their portability. NIRS is relatively inexpensive, has excellent temporal resolution and is less prone to artifacts from subject motion. It also allows simultaneous monitoring of relative changes in tissue blood flow and oxygenation [12]. However, the method's spatial resolution is limited and can only provide microvascular function in relatively superficial tissues. In addition, the tissue response to light depends on skin color, body fat and muscle layers, leading to large inter-subject variations [20]. US measurements of FMD in the brachial artery have generated substantial interest in clinical research since its introduction [21]. Numerous studies have since demonstrated the prognostic value of brachial artery FMD [22,23]. However, the methodology suffers from substantial inter- and intra-observer variability [9,24] in that probe placement or US settings such as dynamic range, gain and probe distance can significantly affect diameter measurements [25]. These sources of error are of particular concern since the average relative change of FMD is on the order of 5% only [26].

CMR has also demonstrated its potential for evaluating vascular reactivity. CMR flow velocimetry can be performed with high spatial and temporal resolution as demonstrated in a recent study in which post-occlusion hyperemia in the femoral artery was evaluated in PAD patients and healthy subjects 1.5 T [16]. The implementation of the method requires spatially-selective rf pulses which are not widely available and migration to systems with higher field strength may be challenging due to increased field inhomogeneity. Alternatively, microvascular function can be assessed by quantifying post-occlusive perfusion with arterial spin labeling (ASL) [17] in calf muscle. The ASL-approach is model-dependent and the temporal resolution is limited.

All of the above methods only target one aspect of the vascular reactivity, e.g. hemodynamics, tissue oxygenation, or FMD, as possible indicators of endothelial dysfunction. We posit that quantification of several physiological parameters may lead to a more sensitive assessment for detecting early stage of EDF. As a step toward an integrated CMR protocol to detect early stages of EDF and to extend a MR procedure developed previously based on using oxygen saturation as a dynamic tracer [18], we describe an interleaved multi-echo gradient-recalled (GRE) sequence that is able of temporally resolve venous blood oxygen saturation and flow velocity simultaneously during hyperemia. The feasibility and reproducibility of the proposed method is demonstrated in seven young and five older healthy subjects.

## Methods

### Integration of MR susceptometry and projection-based velocity quantification

MR susceptometry relies on the quantification of the HbO_2_-dependent susceptibility difference between intravascular blood and surrounding tissue [27,28]. For the remainder of the manuscript HbO_2 _is defined as the fractional oxygen saturation expressed in percent. The relative susceptibility difference can be derived from a phase difference image between two gradient echoes differing in echo time. Velocity is computed from a phase difference image between interleaved GREs differing in velocity-encoding. The basic idea of integrating MR susceptometry and velocity quantification is to interleave multi-echo GREs with different velocity encoding [27]. However, in order to achieve adequate temporal resolution to map the time-course of hyperemia, the velocity will be quantified with projections [29] while full 2D echoes will be acquired for HbO_2 _quantification.

### Pulse Sequence

**Figure 1:**
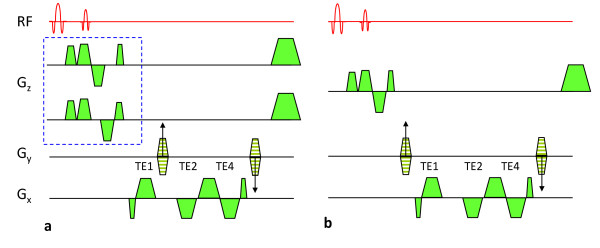
**Multi-echo GRE pulse sequence to **a**) collect velocity-encoded projections (TE1) and full-image echoes**. **b) **Full image is collected at TE1 (note phase encodes are repositioned) for removing tissue signal from the projections. The first RF pulse, followed by a spoiler gradient, is for lipid suppression.

The pulse sequence for temporally-resolved measurement of venous oxygen saturation and flow velocity is shown in Figure 1a. It consists of a fat-suppressed multi-echo GRE pulse sequence for collecting velocity-encoded projections sampled at TE1 (i.e. in the absence of phase-encoding). The same excitation generates two additional echoes (TE2 and TE4), which are phase-encoded, thereby yielding a full image for field mapping. The velocity encoding is toggled after each TR by separating the flow-compensating gradient lobes appropriately (dashed box Figure 1a); the *maximum *temporal resolution is 2 × TR. Thus, while the pulse sequence collects velocity-encoded projections at TE1 the phase-encoding gradient is incremented for the remaining echoes. In this manner, 64 pairs of velocity-encoded projections can be collected while a single 2D field map is obtained from TE2 and TE4. The sequence in Figure 1b collects a 2D reference image at TE1 for removing background tissue signal from the velocity-encoded projections, as explained in more detail below. In addition, data are acquired at TE2 and TE4 for 2D field mapping. In this manner, uninterrupted measurement of HbO_2 _is ensured. We note that TE2 and TE4 have the same first moment in all three directions so that flow-induced phase accumulation is minimized for the field maps.

### Subjects

Seven healthy young (YH, 26 ± 1.6 yrs) and five older healthy (OH, 59 ± 7.3 yrs) subjects without prior history of cardiovascular disease were recruited to evaluate the performance of the interleaved pulse sequence in terms of reproducibility and the potential to detect expected age-related differences between the two groups with parameters of vascular reactivity. To ascertain that all subjects were free of peripheral arterial disease the ankle-brachial index (ABI) was determined. All subjects evaluated had ABI > 0.9. Written informed consent was obtained prior to all examinations following an institutional review board-approved protocol.

### Cuff paradigm

Ischemia was induced by inflating a blood pressure cuff (Aspen Labs A.T.S 1500 Tourniquet System) to 75 mmHg above the systolic pressure in the upper thigh. The location was chosen so as to minimize perturbation of the imaging region (approximately 10 cm superior to the knee) during cuff inflation or deflation. On ten subjects (5 YH and 5 OH), the cuff paradigm consisting of 2 mins baseline, 5 mins occlusion and 6 mins recovery was performed to monitor post-ischemic vascular reactivity.

### Reproducibility

Two YH subjects (not taken from the pool of 5 YH) participated only in the reproducibility study where an abbreviated cuff paradigm (3 mins occlusion and 2 mins recovery) was used in order to allow for three repeated measurements in a single session to assess reproducibility. After each scan the subject was removed from the scanner and allowed to rest for 15 mins to ensure full recovery before repeating cuff occlusion.

### CMR Protocol

All imaging was performed on a 3T Siemens Trio. Phased-array eight-channel knee coil (Invivo Inc., Pewaukee, WI) was used to acquire axial images of femoral vessels. From the scout images an appropriate readout direction was chosen to avoid overlap between the femoral artery and vein along the projection direction. For the data acquisition the following imaging parameters were used: TE/TR = 5.0/39.1 ms, flip angle = 13°, Bandwidth = 520 Hz/pix, voxel size = 1 × 1 × 5 mm^3^, matrix size = 128 × 128. In addition to acquiring velocity-encoded projections taken with equal magnitude but opposite-sign first moments, flow-compensated projections (not shown in Figure 1) were also acquired repeatedly to ensure acquisition of unaliased phase data. Thus, three projections offer the flexibility of achieving either VENC of 125 or 250 cm/s, as needed, i.e. by computing the phase difference between acquisitions taken with opposite polarity flow-encoding gradients or between zero and non-zero first-moment encoded projections. The pulse sequence of Figure 1a was launched 10 s prior to and was run for 60 s after the cuff release. For the remaining recovery period only HbO_2 _was monitored with the flow-compensated pulse sequence (Figure 1b). The pulse sequence achieves temporal resolutions of 5 s and 117 ms for dynamic oximetry and velocity quantification, respectively.

### Data Analysis

#### HbO_2 _quantification

The raw k-space data were processed off-line. A phase difference image was computed with the images of equal polarity echoes, e.g. TE2 and TE4. The effects of low spatial-frequency modulations of static magnetic field were minimized by fitting the data, after appropriate weighting and masking, to a second-order polynomial [30]. The hemoglobin oxygen saturation, HbO_2_, in the femoral vein was computed as $HbO_{2}=\left[1-\frac{6|\text{Δ}\phi |∕\text{Δ}TE}{\gamma \text{Δ}\chi_{do}Hct\cdot B_{o}\left(3\cos^{2}\theta -1\right)}\right]$[1].

where *Δϕ *is the average intravascular phase relative to surrounding tissue, *ΔTE *= *TE4 *- *TE2*, is the inter-echo, *γ *is the gyromagnetic ratio of water protons, Δ*χ_do _*= 4*π *× (0.27 ± 0.02)*ppm *[31] is the susceptibility difference between fully deoxygenated and fully oxygenated erythrocytes in SI units, *θ *is the vessel tilt angle relative to the main field *B_o _*and *Hct *(hematocrit) is the volume fraction of erythrocytes in packed blood.

The time-course of HbO_2 _characterize vascular reactivity with three parameters: *washout time*, *upslope *and *overshoot*, as described previously [18]. The washout time refers to the elapsed time to observe oxygen-depleted capillary blood at the measurement location after cuff deflation (t = 0). Resaturation occurs at a mean rate termed *upslope *and the transient increase in blood flow rate leads to an *overshoot*, an above-baseline HbO_2 _level.

#### Blood velocity quantification

In order to isolate the vascular signal from static tissue a projection derived from the reference image is subtracted from the velocity-encoded projections as described previously [29]. In brief, the vessels of interest are masked out manually from the complex reference image and then Fourier transformed to k-space. The projection, which contains tissue signal only, is then subtracted from the velocity-encoded projections, thus isolating blood signal. The subtraction is performed in the complex domain to retain the phase information. The phase differences between velocity-encoded projections are converted to velocity using the relation between phase *ϕ *and velocity *v*, as *ν *= *ϕ·VENC/π*. The spatially averaged velocities of the vessels of interest are derived by averaging the velocity along the readout direction within the vessel boundaries. Thus, at each temporal point, the average velocity represents the velocity averaged over the lumen.

The following parameters are derived from the time-resolved blood flow velocity: time required to reach peak average velocity (*time-to-peak *(*TTP*)), the *duration of forward flow *(*T_ff_*) and *maximum change in the flow rate *relative to baseline (*ΔQ_max_*), which we define as the ratio, *v_peak, avg/_v_base, avg_*. The parameter *v_base, avg _*is computed by averaging the time-resolved velocity over 6 - 8 cardiac cycles and *v_peak, avg _*is the peak of the temporally averaged velocity via 2-second sliding window during the course of hyperemia. Computation of *ΔQ_max _*ignores the very small change in lumen area that is not detectable at the resolution of 1 mm. This leads to an error no greater than 5% based on previous observations [16,32] where the lumen diameter was found to increase by 3-5% during peak hyperemia.

## Results

**Figure 2:**
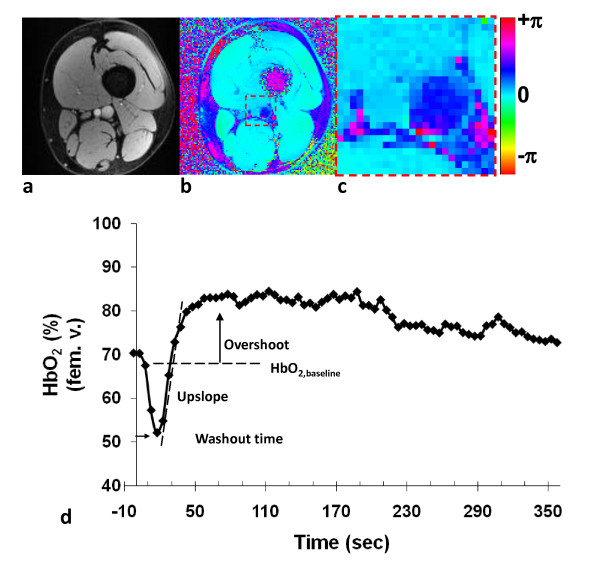
**a) Sample magnitude and b) phase difference image for quantifying venous HbO_2 _in the femoral vein of a healthy young subject**. The region in the dashed box is magnified in panel **c**). **d**) Representative time-course of HbO_2_.

**Figure 3:**
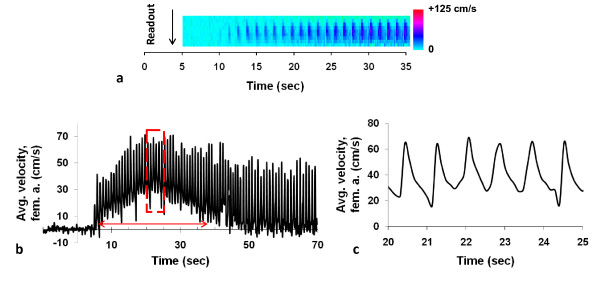
**Post-occlusive blood flow velocity in the femoral artery in a 25 year-old male**. **a**) Magnified view of a velocity image reconstructed from velocity-encoded projections during the early phase of hyperemia after the cuff deflation (background cropped). The time axis and the readout direction are indicated and each vertical line represents a velocity profile obtained from a pair of velocity-encoded projections. **b**) time-course of blood flow velocity (region of red rectangle magnified in panel **c**). The duration of forward flow T_ff _is indicated by the double arrow. The magnification in **c**) shows that each spike corresponds to a systolic peak.

Representative magnitude and phase difference images of the femoral artery and vein are shown in Figure 2. The phase difference image (Figure 2b) demonstrates the susceptibility-induced phase contrast between vein and artery. Figure 2c more clearly shows that the relative difference in the magnetic susceptibility is negligible between tissue and fully oxygenated arterial blood. The temporal position of HbO_2 _time-course in femoral vein (Figure 2d) is defined with respect to the acquisition time of the center of k-space, e.g. 2.5 s, 7.5 s, 12.5 s etc. According to error propagation (eqn. 1) the predominant contributor to standard error of HbO_2 _is the error in Δϕ; conservatively estimated at ± 3%HbO_2_. During baseline the blood oxygen saturation level is approximately 67% (dashed horizontal line). In healthy subjects, the observed blood oxygenation at the imaging slice typically ranges from 30 to 85% post occlusion, representing the saturation level of washed-out oxygen-depleted blood and maximum above-baseline value during peak hyperemia, respectively. The range of HbO_2 _values qualitatively agrees with tissue HbO_2 _measurements made with near-infrared spectroscopy [12]. Figure 3 shows time-resolved velocity data derived from projection images of the femoral artery. Figure 3a displays time series of projection images, Figure 3b the time-course of velocity during hyperemia (expanded in Figure 3c). The latter highlights the sharp increase in the averaged blood flow velocity and monophasic waveform (absence of retrograde flow) during the early phase of hyperemia, presumably due to reduced microvascular resistance. The velocity waveforms as well as the typical peak average velocity agree with previous MR quantifications [15,33]. The results are summarized in Table 1 where the average value and standard deviation (SD) for each parameter are reported for each group. Uncertainty (determined via error propagation) for each parameter is also included under each column heading. Based on a two-tailed t-test, assuming unequal variances, overshoot and TTP were significantly different (p < 0.05) between YH and OH subjects. We also observe that the standard deviations of the parameters (all but the overshoot) are greater among the OH, which may reflect longer exposure to different types of lifestyles.

**Table 1:** Summary of physiological parameters derived from dynamic oximetry and velocimetry.

Age (yrs)	Washout (± 2.5 sec)	Upslope (± 0.2%HbO_2_/sec)	Overshoot (± 3%HbO_2_)	ΔQ_max_ ± 0.5	T_ff_ (± 1 sec)	TTP (± 1 sec)
**Old **(59 ± 7.3 yrs)						
58	27.5	0.49	2	3.8	28	20
55	22.5	0.78	7	10.4	25	16
56	12.5	1.9	7	8.3	30	16
55	17.5	0.9	10	13.8	15	14
72	17.5	1.3	10	5.9	26	17
**59.2 ± 7.3**	**19.5 ± 5.7**	**1.07 ± 0.55**	**7.2 ± 3.3**	**8.44 ± 3.9**	**24.8 ± 5.8**	**16.6 ± 2.2**
**Young **(26 ± 1.6 yrs)						
25	17.5	1.2	13	9.2	22	14
26	17.5	1.8	25	12	25	14
26	12.5	1.8	30	12.5	30	11
25	12.5	1.9	25	7.7	20	11
29	12.5	1.9	20	6.65	25	14
**26.2 ± 1.6**	**14.5 ± 2.7**	**1.72 ± 0.29**	**22.6 ± 6.4**	**9.61 ± 2.6**	**24.4 ± 3.8**	**12.8 ± 1.64**
*p-value	**0.127**	**0.058**	**0.003**	**0.592**	**0.9**	**0.017**

*Two-tailed t-test assuming unequal variances.

**Figure 4:**
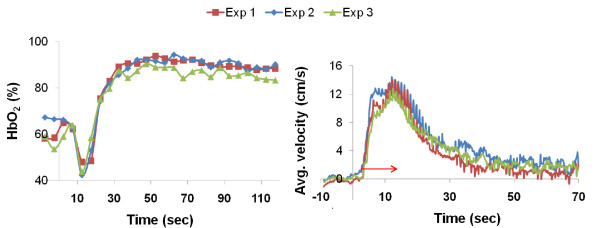
**Post-occlusive time-courses of a) HbO_2 _in femoral vein and b) average blood flow velocity in femoral artery after 3 mins of cuff occlusion**. The red arrow indicates time-to-peak (TTP), the temporal location of the peak temporally-averaged velocity over a 2-second sliding window.

Repeated measurements of HbO_2 _(Figure 4a**) **and blood flow velocity (Figure 4b) are shown for one of the subjects. The largest variations between scans in the HbO_2 _time-course typically occur just prior to cuff release due to the absence of flow-related enhancement. However, this is largely irrelevant since none of the parameters of interest are affected by the pre-cuff-release signal course. In Figure 4b the blood flow velocity was averaged over 2 s sliding-window intervals for comparing the velocity time-courses of three measurements. The average oximetric and velocimetric parameters from the three successive scans along with their SD and coefficients of variation (CV) are summarized in Table 2. For the reproducibility study, the average blood flow velocity at rest was not quantified (therefore ΔQ_max _could not be computed). Instead, we report peak average velocity, v_peak, avg_, (Figure 4b). For both subjects the average CV is approximately 8% but the CV was as high as 16% (overshoot in 26 yr-old subject). However, it should be noted that the intersubject SD is about 50% and thus much greater than intrasubject SD (Table 1) for all parameters.

**Table 2:** Repeat measurements of reactive hyperemia in two healthy subjects.

	Washout (sec)	Upslope (HbO_2_/sec)	Overshoot (HbO_2_)	T_ff_ (sec)	TTP (sec)	v_peak, avg_ (cm/s)
**Healthy 39 yr-old**						
Exp 1	15	2	30	19	10	13.5
Exp 2	12.5	1.9	32	15	9	13.6
Exp 3	12.5	2.2	30	17	9	12.5
Avg	13.3	2.0	30.7	17	9.3	13.2
Std	1.4	0.15	1.15	2	0.58	0.61
CV (%)	10.5	7.5	3.7	11.8	6.2	4.6
**Healthy 26 yr-old**						
Exp 1	22.5	1.32	11	10	4.1	20.6
Exp 2	22.5	1.29	8	12	4.4	24.6
Exp 3	22.5	1.54	10	12	4	25.4
Avg	22.5	1.38	9.7	11.3	4.17	23.5
Std	0	0.136	1.53	1.15	0.21	2.57
CV (%)	0	9.9	15.8	10.2	5.0	10.9

## Discussion

The work shows that simultaneous measurement of dynamic oximetric and arterial flow velocity is possible for assessing post-occlusive peripheral vascular reactivity. In contrast, a sequential measurements would require application of the cuff twice, which would not be tolerable by a significant number of patients. The method returns six parameters, three each related to the time-course of venous HbO2, and arterial flow velocity. The time-course of HbO_2 _(referred to as dynamic oximetry) follows the fate of the oxygen-depleted blood in the capillary bed upon restoration of flow and its subsequent replacement by normally oxygenated venous blood. It thus provides quantitative information on how rapidly oxygen-depleted blood is washed out of tissue as well as the rate at which tissue is resaturated. In addition, the overshoot may reflect the magnitude of hyperemia since tissue has reduced time to extract O_2_, a time-consuming process driven by perfusion, thus greater above-baseline HbO_2 _is expected with more pronounced hyperemia. The oximetric parameters derived in this pilot study are consistent with those of the authors' prior work in which a significantly greater overshoot was observed in healthy young compared to older subjects [18].

The velocity profile of the healthy subjects qualitatively agrees with previous "real-time" phase-contrast CMR data [15,16] including the duration of forward flow (T_FF_; 20 - 35 s) and peak average velocity (~ 40 - 60 cm/s). The unique feature of the "real-time" techniques [15,16], including the current method, is the ability to resolve the velocity waveform for each cardiac cycle during hyperemia. In all cases, the blood flow direction does not reverse during the early phase of hyperemia (Figure 3b) due to significant reduction in microvascular resistance. Since vasodilation is generally triggered by NO release, TTP and T_ff _may assess the microvascular function's response to an ischemia, i.e. the rate and maximum bioavailability of nitric oxide release.

The projection method has the usual limitation when a spatial dimension is sacrificed for improved temporal resolution in that velocity is resolved along a single spatial direction only and thus inherently averaged along the projected lumen. Hence, peak velocity cannot be quantified but rather an average in the projection direction is extracted. Lastly, "misregistration" of femoral vessels between reference image and projections during hyperemia is largely avoided by applying the cuff 10 to 15 cm from the imaging slice, and since the blood velocity near the vessel wall is nearly zero the error in average velocity is expected to be small.

The proposed method is a component of an ongoing development of CMR-based techniques for measuring multiple parameters across different vascular territories as part of a single examination to study endothelial dysfunction.

## Conclusions

The quantitative imaging method introduced provides a series of parameters on peripheral vascular reactivity in about fifteen minutes, allowing time for additional quantification of physiological parameters in a single scan session and should be of value to study preclinical cardiovascular disease.

## Competing interests

The authors declare that they have no competing interests.

## Authors' contributions

ML conceived, designed and implemented the pulse sequence, collected and analyzed the data. FW participated in the design of experiment and study, and helped to draft the manuscript. All authors read and approved the final manuscript.
